# Immunomodulatory effects of bacteriocinogenic and non-bacteriocinogenic *Lactococcus cremoris* of aquatic origin on rainbow trout (*Oncorhynchus mykiss*, Walbaum)

**DOI:** 10.3389/fimmu.2023.1178462

**Published:** 2023-04-20

**Authors:** Diogo Contente, Patricia Díaz-Rosales, Javier Feito, Lara Díaz-Formoso, Félix Docando, Rocío Simón, Juan Borrero, Pablo E. Hernández, Patrícia Poeta, Estefanía Muñoz-Atienza, Luis M. Cintas, Carolina Tafalla

**Affiliations:** ^1^ Grupo de Seguridad y Calidad de los Alimentos por Bacterias Lácticas, Bacteriocinas y Probióticos (SEGABALBP), Sección Departamental de Nutrición y Ciencia de los Alimentos, Facultad de Veterinaria, Universidad Complutense de Madrid, Madrid, Spain; ^2^ Fish Immunology and Pathology Laboratory, Animal Health and Research Center (CISA), National Institute for Agricultural and Food Research and Technology (INIA), Spanish National Research Council (CSIC), Madrid, Spain; ^3^ Microbiology and Antibiotic Resistance Team (MicroART), Department of Veterinary Sciences, Universidade de Trás-os-Montes e Alto Douro (UTAD), Vila Real, Portugal

**Keywords:** fish, probiotics, lactic acid bacteria, bacteriocins, nisin, immunomodulation

## Abstract

Lactic Acid Bacteria (LAB) are a group of bacteria frequently proposed as probiotics in aquaculture, as their administration has shown to confer positive effects on the growth, survival rate to pathogens and immunological status of the fish. In this respect, the production of antimicrobial peptides (referred to as bacteriocins) by LAB is a common trait thoroughly documented, being regarded as a key probiotic antimicrobial strategy. Although some studies have pointed to the direct immunomodulatory effects of these bacteriocins in mammals, this has been largely unexplored in fish. To this aim, in the current study, we have investigated the immunomodulatory effects of bacteriocins, by comparing the effects of a wild type nisin Z-expressing *Lactococcus cremoris* strain of aquatic origin to those exerted by a non-bacteriocinogenic isogenic mutant and a recombinant nisin Z, garvicin A and Q-producer multi-bacteriocinogenic strain. The transcriptional response elicited by the different strains in the rainbow trout intestinal epithelial cell line (RTgutGC) and in splenic leukocytes showed significant differences. Yet the adherence capacity to RTgutGC was similar for all strains. In splenocyte cultures, we also determined the effects of the different strains on the proliferation and survival of IgM^+^ B cells. Finally, while the different LAB elicited respiratory burst activity similarly, the bacteriocinogenic strains showed an increased ability to induce the production of nitric oxide (NO). The results obtained reveal a superior capacity of the bacteriocinogenic strains to modulate different immune functions, pointing to a direct immunomodulatory role of the bacteriocins, mainly nisin Z.

## 1 Introduction

As the fastest-growing agri-food sector worldwide, aquaculture is recognized as a keystone in global animal protein supply, food security and nutrition. To meet this growing demand, the aquaculture sector requires intensification, expansion, and modernization. In this regard, the Food and Agriculture Organization (FAO) has outlined the vision and goals of the Blue Transformation 2022-2030, aiming for the sustainable development of the sector in the future (1). Often this swift intensification in the farming system has been followed by declines in biosecurity, hygiene, animal welfare or husbandry standards. Consequently, the increasing emergence and incidence of infectious disease outbreaks has led to growing economic losses. This situation has promoted the widespread use of antimicrobial drugs in aquaculture. However, the indiscriminate use of these antibiotics promotes the occurrence of antimicrobial-resistant bacteria, which not only contribute to the contamination of the aquatic environments and sediments, but also pose a serious global threat to both animal and human health (2–4). Thus, several countries and multilateral institutions, such as the European Union, have restricted and heavily legislated the use of antibiotics in aquaculture, aiming towards more eco-friendly and sustainable rearing practices (2, 5).

In this regard, the use of probiotics is deemed as a reliable alternative to traditional chemotherapy, with well-established and promising results in several reared species. Research on probiotics in aquaculture has been regularly focused on studying their antimicrobial properties, such as the production of organic acids, hydrogen peroxide, lysozymes, lytic enzymes, and antimicrobial peptides (referred to as bacteriocins). Nevertheless, their mechanisms of action are far broader, including competition for adhesion sites, improved tolerance to stress, host nutrition enhancement, and/or improvement of the host innate and acquired immune responses (6–8).

Lactic Acid Bacteria (LAB), which are generally classified with the Qualified Presumption of Safety (QPS) status, are the group of bacteria most frequently proposed as probiotics for aquaculture. In this respect, the production of bacteriocins by LAB is a common trait thoroughly documented, being regarded as a key probiotic antimicrobial strategy (9–11). Additionally, several LAB species of aquatic origin have been associated with positive immunostimulatory results, amongst them *Leuconostoc* spp., *Pediococcus* spp. and *Weissella* spp., with particular focus on the genera *Lactobacillus* and *Lactococcus*. Nonetheless, the immunomodulatory effects of bacteriocinogenic-LAB and the precise immunostimulatory role of bacteriocins have rarely been assessed in aquaculture (10–16).

In previous studies, our group isolated and characterized a nisin Z (NisZ)-producing strain, *Lactococcus cremoris* WA2-67, from the rearing tank of a Spanish rainbow trout (*Oncorhynchus mykiss*) farm, which demonstrated a strong *in vivo* protective effect against *Lactococcus garvieae* infection on rainbow trout. This protective effect was mainly attributed to the production of NisZ (10, 11). Nisin A (NisA) is a 34 amino acid-long lantibiotic, and it is by far the most well described bacteriocin, with applications in human and veterinary medicines, as well as in the food technology sector. To date, several natural variants of NisA differing in 1-2 amino acids have been described, such as nisin F, Q, U and Z, being the latter the most widespread natural variant (11). In this study, we aimed to evaluate the immunomodulatory effects of bacteriocins in rainbow trout by comparing the effects exerted by *L. cremoris* WA2-67 (10, 11), to those provoked by a recombinant multi-bacteriocinogenic strain (nisin Z, and garvicin Q and A producer), *L. cremoris* WA2-67 (pJFQIAI) (Feito et al., unpublished results), and a non-bacteriocinogenic isogenic mutant (*L. cremoris* WA2-67 Δ*nisZ*) previously generated by our group (11). Initially, we analyzed the modulatory effects of these three *L. cremoris* strains on the rainbow trout intestinal epithelial cell line RTgutGC by studying the transcriptional response of a range of genes, including those related to the expression of antimicrobial peptides (AMPs), and to the intestinal barrier integrity and homeostasis. We also evaluated the adherent capacity of the three lactococcal strains to this cell line. Additionally, we assessed the immunomodulatory effects on rainbow trout splenic leukocytes performing a transcriptional analysis, analyzing the effects on IgM^+^ B cells and determining the effects of the bacteria on the respiratory burst activity and nitric oxide (NO) production. The data provided hereby further support the immunomodulatory potential of aquatic bacteriocinogenic-LAB and demonstrate the involvement of bacteriocins, namely of NisZ, in some of these immunomodulatory effects.

## 2 Materials and methods

### 2.1 Bacterial strains growth conditions

Three *L. cremoris* strains of aquatic origin were used in this work. The wild-type bacteriocinogenic strain (*L. cremoris* WA2-67) and the non-bacteriocinogenic isogenic strain (*L. cremoris* WA2-67 Δ*nisZ*), were grown in the Man, Rogosa and Sharp broth (MRS, Basingstoke, UK) and incubated at 30°C overnight (11). On the other hand, the recombinant multi-bacteriocinogenic strain previously generated by our group, *L. cremoris* WA2-67 (pJFQIAI), was grown in MRS supplemented with chloramphenicol at a concentration of 5 μg/ml (Sigma-Aldrich Inc., USA), under the same incubation conditions (Feito et al., unpublished results).

### 2.2 Cell lines

The rainbow trout intestinal epithelial cell line RTgutGC used in this work was routinely maintained at 20°C, cultured in Leibovitz medium (L-15, Invitrogen, USA), supplemented with an antibiotic mixture consisting of 100 I.U./ml penicillin and 100 µg/ml streptomycin (P/S, Life Technologies, USA) and 10% fetal calf serum (FCS, Life Technologies) (17, 18). Cells were regularly maintained in flasks of 75 cm^2^, and at confluence were split into two new 75 cm^2^ flasks, which would regularly be confluent again after approximately one week. Confluent cells were washed with the medium without antibiotics and detached with trypsin (Invitrogen).

### 2.3 Experimental fish

Healthy individuals of rainbow trout (*Oncorhynchus mykiss*) of approximately 100 g were obtained from the Cienfuentes fish farm (Cifuentes, Guadalajara, Spain). Fish were maintained in an aerated recirculating system at 15°C, exposed to a photoperiod of 12:12 h light/dark, and were fed twice a day with a commercially available feed (Skretting, Spain). Fish were acclimatized to laboratory conditions for at least two weeks prior to any experimental procedure. During this period, no clinical signs of disease were observed. The experiments performed during this work complied with the Guidelines of the European Union Council (2010/63/EU) for the use of laboratory animals and have been approved by the INIA Ethics Committee (Code PROEX 065.3/21).

### 2.4 Splenic leukocyte isolation

Rainbow trout of approximately 100 g were sacrificed by benzocaine (Sigma-Aldrich Inc.) overdose by immersion (50 mg/ml) to isolate the spleen (19). Single cell suspensions were obtained by passing the spleen through a 100 µm nylon cell strainer (BD Biosciences, USA) using L-15 medium supplemented with P/S, 10 U/ml of heparin (Sigma-Aldrich) and 2% FCS. The suspensions were then placed onto 30/51% discontinuous Percoll (GE Healthcare, USA) density gradients and centrifuged at 500 x *g* for 30 min at 4°C without brake. Cells at the interface were collected and washed with L-15 supplemented with antibiotics and 5% FCS. The viable cell concentration was analyzed by trypan blue exclusion (Sigma-Aldrich), and cells were finally resuspended in L-15 with 5% FCS at a concentration of 2 x 10^6^ cells/ml.

### 2.5 Gene expression analysis

RTgutGC cells seeded in 24-well plates (1 x 10^6^ cells per well) were incubated with1 x 10^6^ cfu/ml of each of the three bacterial strains at 20°C for 24 h. Isolated spleen leukocytes were also distributed in 24-well plates at a concentration of 2 x 10^6^ cells/ml (1 ml per well), and then incubated with 1 × 10^6^ cfu/ml of each of the three bacterial strains at 20°C for 24 h. Untreated wells with no bacteria were included as controls in both experiments.

In all cases, after the incubation time, media was removed, and total RNA extracted from cells using TRI Reagent solution (Invitrogen) according to the manufacturer instructions. RNA was then quantified using a NanoDrop 1000 Spectrophotometer (Thermo Fisher Scientific) and treated with DNase to remove remnants of genomic DNA that might interfere with the PCR reactions. Subsequently, cDNA was obtained from 1 µg of total RNA using the RevertAid Reverse Transcriptase (Thermo Fisher Scientific), primed with oligo(dT)_23_VN, following the manufacturer´s instructions. To evaluate gene transcription levels, real-time PCR was performed in a LightCycler96 System (Roche, Switzerland) using FastStart Essential DNA Green Master reagents (Roche) and specific primers (**Table S1**). Each sample was exposed to the following conditions: 10 min at 95°C, followed by 40 amplification cycles (10 s at 95°C, 10 s at 60°C and 10 s at 72°C). A dissociation curve was obtained by reading fluorescence every degree between 60°C and 95°C to ensure only a single product had been amplified. The relative expression levels of the genes were normalized to the expression of control housekeeping genes, following the MIQE guidelines (20), using β-actin (*bactin*) for splenic leukocytes, and the elongation factor 1 alpha (*ef1a*) for RTgutGC. These housekeeping genes were selected after verifying that no statistical differences were detected among their Ct values from different samples. Expression levels were calculated using the 2^-ΔCt^ method, being ΔCt determined by subtracting the housekeeping gene value from the target cycle threshold. Negative controls with no template and *minus*-reverse transcriptase controls were included in every case.

### 2.6 Adherence assay

The adherence assay was performed according to a method previously described (21). Shortly, the grown *L. cremoris* strains were centrifuged at 3800 x *g* for 5 min at 4°C and washed in 0.9% NaCl. Bacteria were labelled with the SYTO™ BC Green Fluorescent Nucleic Acid Stain (Thermo-Fisher) according to the manufacturer instructions and incubated in the dark at room temperature (RT) for 30 min. Subsequently, they were centrifuged at 3800 x *g* for 5 min at 4°C and re-suspended in L-15 containing 10% FCS.

To test the adherence of *L. cremoris* strains to the RTgutGC cell line, cells were seeded in 24-well plates at 5 x10^5^ cells/ml (1 ml per well) and incubated at 20°C for 24 h in L-15 supplemented with 10% FCS. At this point, cells were exposed to 100 μl of the previously labeled bacteria (final concentration 1 x 10^7^ cfu/ml) and incubated at 20°C for 24 h. RTgutGC cells not incubated with the bacteria and cells exposed to unlabeled bacteria were also included as negative controls. After the incubation period, cells were washed twice with 0.9% NaCl to remove non-adherent bacteria. Afterwards, trypsin was added to each well to detach the cells, which were subsequently washed twice with staining buffer (phenol red-free L-15 medium supplemented with 2% FCS) and analyzed on a FACS Celesta™ flow cytometer equipped with BD FACSDiva software (BD Biosciences). The flow cytometry analysis was performed with FlowJo^®^ v.10 (FlowJo LLC).

### 2.7 Effect of bacteria on IgM^+^ B cell survival

Isolated splenic leukocytes distributed into 96-well plates were exposed to 1 x 10^6^ cfu/ml of each of the cultured bacterial strains. Controls without bacteria were also included in the experiment. After 48 h of incubation at 20°C, the percentage of IgM^+^ cells in the cultures was assessed by flow cytometry. For this, leukocytes were incubated with a specific monoclonal antibody (mAb) against rainbow trout IgM [1.14 mAb mouse IgG_1_ coupled to R-phycoerythrin (R-PE), 1 µg/m], at 4°C for 1 h in staining buffer. The monoclonal antibody was fluorescently labeled using the R-PE Lightning-Link labeling kit (Innova Biosciences, UK) following the manufacturer instructions. After the incubation, leukocytes were washed twice with staining buffer and counter-stained with 0.2 μg/ml 4′,6-diamidino-2-phenylindole (DAPI, Sigma-Aldrich) to distinguish dead cells and discard them from the analysis. An isotype control was used in parallel to discard unspecific binding of the mAb. Finally, the samples were analyzed on a FACS Celesta™ flow cytometer equipped with BD FACSDiva software. Flow cytometry analysis was performed with the software FlowJo^®^ v.10.

### 2.8 Proliferation of IgM^+^ B cells

The Click-IT^®^ EdU Alexa Fluor^®^ 488 Flow Cytometry Assay Kit (Life Technologies) was used to measure the proliferation of splenic IgM^+^ B cells in response to the different bacterial strains. Briefly, splenic leukocytes, distributed into 96-well plates at a concentration of 2 x 10^6^ cells/ml, were incubated with the three cultured bacterial strains at a concentration of 1 x10^6^ cfu/ml at 20°C for 72 h. Cells not exposed to bacteria were included as controls. Subsequently, 1 μM EdU (5-ethynyl-2′-deoxyuridine) was added to the cultures, followed by another incubation of 24 h. The cells were then collected, and their viability assessed prior to fixation and permeabilization using the LIVE/DEAD™ Fixable Near-IR Dead Cell Stain kit (Invitrogen) for 30 min, following the kit´s instructions. Thereafter, cells were washed and stained with anti-IgM (1.14 mAb mouse IgG_1_ coupled to R-PE, 1 µg/m) at 4°C for 30 min. Lastly, cells were fixed, permeabilized, and incubated with specific reagents to detect the incorporation of EdU to the DNA of proliferating B cells following the manufacturer´s instructions. Samples were then analyzed by flow cytometry as described above.

### 2.9 Respiratory burst activity

Leukocyte respiratory burst activity was assayed via the reduction of the ferricytochrome c by released superoxide anion (O_2_-). Briefly, splenic leukocytes were distributed into 96-well plates at a concentration of 2 x 10^6^ cells/ml (100 µl per well). After an overnight incubation, the media was removed by centrifugation (500 x *g* for 5 min) and the cells in each well stimulated with 100 µl of HBSS (Hank’s Balanced Salt Solution, Gibco) containing 1 x 10^4^ cfu/ml of each of the three bacterial strains and ferricytochrome c (2 mg/ml, Sigma). After 30 min at RT in darkness, the optical density (OD) was measured at 550 nm in a multiscan spectrophotometer FLUOstar Omega (BMG Labtech, Germany).

### 2.10 Nitric oxide (NO) production

Splenic leukocytes were distributed in 96-well plates at a concentration of 4 x 10^5^ cells/ml (200 μl per well), and then incubated independently with 1 x 10^4^ cfu/ml of each of the three cultured bacterial strains at 20°C for 48 h. Cells not exposed to bacteria were included as controls. At this point, 50 μl of each well were transferred to a new 96-well plate to which 100 μl of 1.0% sulfanilamide in 2.5% H_3_PO_4_ were added followed by 100 μl of 0.1% N-naphthyl-ethylenediamine in 2.5% H_3_PO_4_. The plates were then incubated for 5 min at RT in darkness. Lastly, the absorbance was recorded at 540 nm using a FLUOstar Omega (BMG Labtech, Germany). Internal positive and negative controls were included in all assays.

### 2.11 Statistical analysis

Data curation, statistical analyses and graphical representations were performed using the GraphPad Prism 8 software (GraphPad Software, San Diego, California, USA). All data were verified for normal distribution using the Shapiro-Wilk test and transformed when required by Napierian logarithm. Statistical analyses were then performed using paired Student’s *t*-tests. Additionally, in all experiments, one-way ANOVAs were performed to compare data between different bacterial treatments.

## 3 Results

### 3.1 Transcriptional effects of the bacterial strains on RTgutGC cells

The immunostimulatory effects of the three *L. cremoris* strains were first studied in the RTgutGC intestinal epithelial cell line, determining the effects of the bacteria on the transcriptional response of these cells. We assessed the levels of transcription of different pro-inflammatory cytokines such as tumor necrosis factor α (*tnfa*), interleukin 1β (*il1b*) and interleukin 8 (*il8*); an anti-inflammatory cytokine (*il10*), and different AMPs (*cathelicidin 2* and *hepcidin*). Moreover, we analyzed the levels of transcription of multiple intestinal barrier integrity and homeostasis related genes. These included genes coding for the tight junction genes e-cadherin (*cdh1*) and claudin 3 (*cldn3*), zonula occludens (*zo1*) an actin-binding protein associated with the villi structures (*villin*) and a gene responsible for intestinal mucin production (*imuc*) (18, 22).

**Figure 1:**
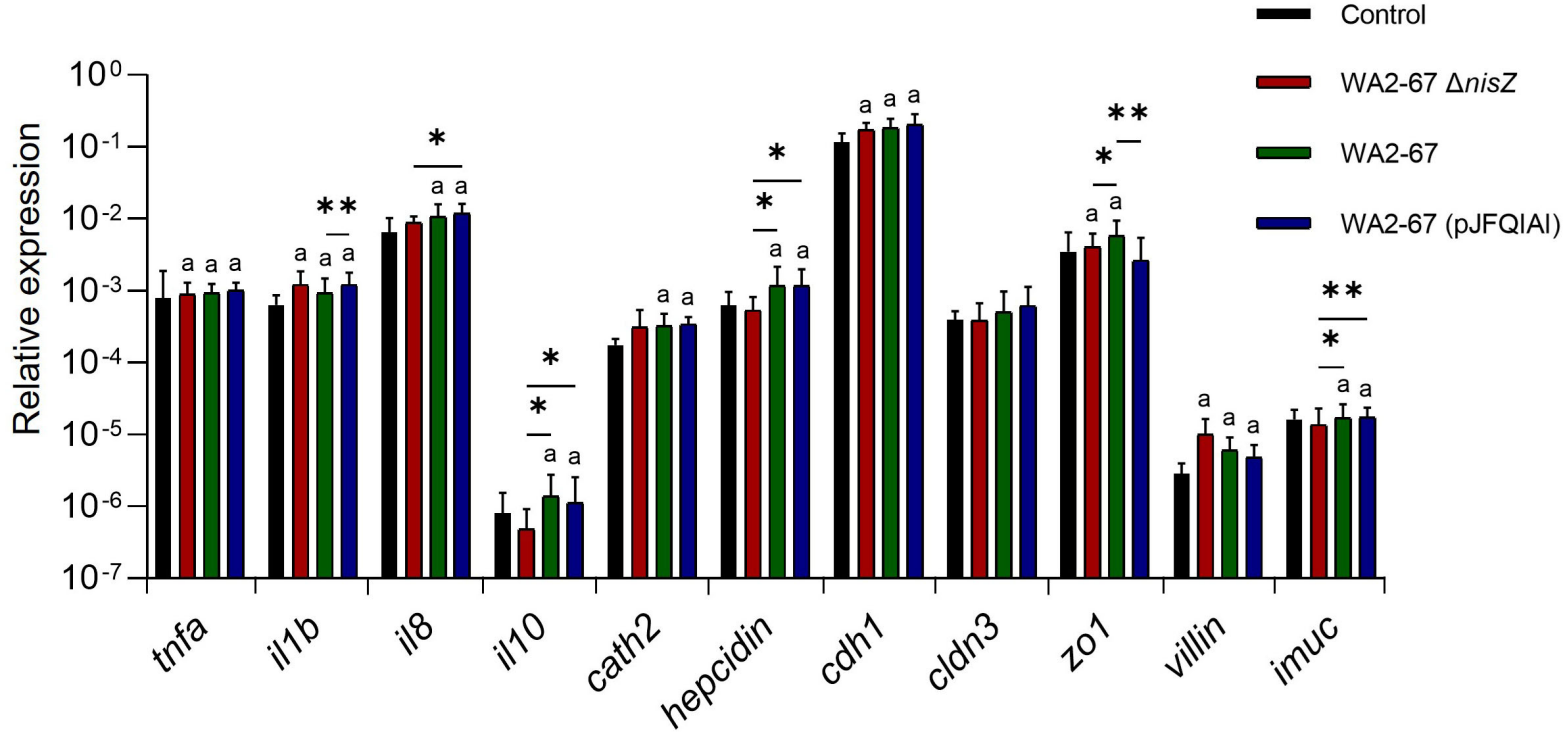
Transcriptional response of RTgutGC cells to different LAB strains: *L. cremoris* WA2-67 Δ*nisZ*, *L. cremoris* WA2-67 and *L. cremoris* WA2-67 (pJFQIAI). RTgutGC cells were incubated with 1 x 10^6^ cfu/ml of each bacterial strain at 20°C for 24 h. Subsequently, RNA was extracted and the levels of transcription of different genes analyzed by real-time PCR. Relative gene expression levels were normalized to the transcription of the housekeeping gene *ef1a*. Data are shown as mean fold change + SD (n = 8). The letter (a) represents transcription levels significantly different than those observed in cells not exposed to bacterial strains (control), while the asterisks indicate levels significantly different between bacterial treatments as indicated (*p ≤ 0.05 and **p ≤ 0.01).

Our results showed that while all LAB upregulated the transcription of two of the pro-inflammatory cytokines tested (*il1b* and *tnfa*), only the bacteriocinogenic strains were capable of significantly upregulating *il8* transcription (**Figure 1**). Likewise, only the two bacteriocinogenic *L. cremoris* significantly increased the expression levels of the anti-inflammatory cytokine *il10*, with statistically significant differences when compared with both control cells and those exposed to *L. cremoris* Δ*nisZ* (**Figure 1**). Similarly, only the bacteriocinogenic LAB upregulated the expression of AMPs (*cathelicidin 2* and *hepcidin*) (**Figure 1**). Concerning the intestinal barrier tight junction genes, all LAB significantly upregulated the expression of *cdh1* and *villin* (**Figure 1**). Moreover, both *L. cremoris* WA2-67 and *L. cremoris* Δ*nisZ* significantly increased the expression levels of *zo1*, with the wild type strain demonstrating statistically significant results in comparison to all other treatments (**Figure 1**). Finally, the expression of *imuc* was only significantly stimulated in response to the bacteriocinogenic strains, in both cases reaching values significantly higher than those obtained in response to the non-bacteriocinogenic strain (**Figure 1**).

### 3.2 Adherence to RTgutGC cells

**Figure 2:**
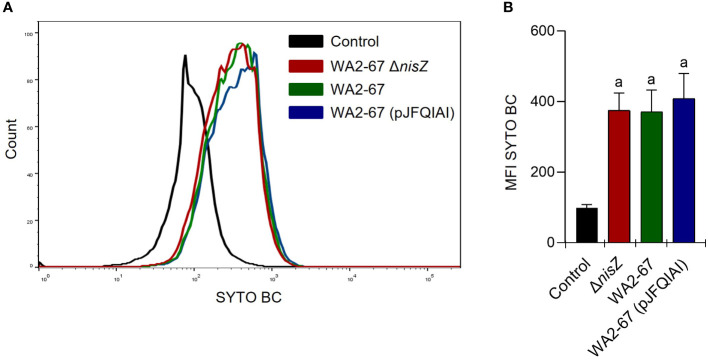
Adherence of *L. cremoris* strains to RTgutGC cells. RTgutGC cells were incubated with the different strains at 20°C for 24 h. Control cells without bacteria were similarly treated. Adherence was then estimated by flow cytometry. Representative histograms **(A)** are shown along with a graph **(B)** representing the mean values of MFI calculated for each peak, shown as mean + SD (n = 12). The letter (a) represents adherence values significantly different to control cells.

All three LAB strains showed a strong ability to adhere to the intestinal epithelial cell line RTgutGC. However, no significantly different binding capacities were found among the different strains (**Figure 2**).

### 3.3 Transcriptional effects of the bacterial strains on splenic leukocytes

**Figure 3:**
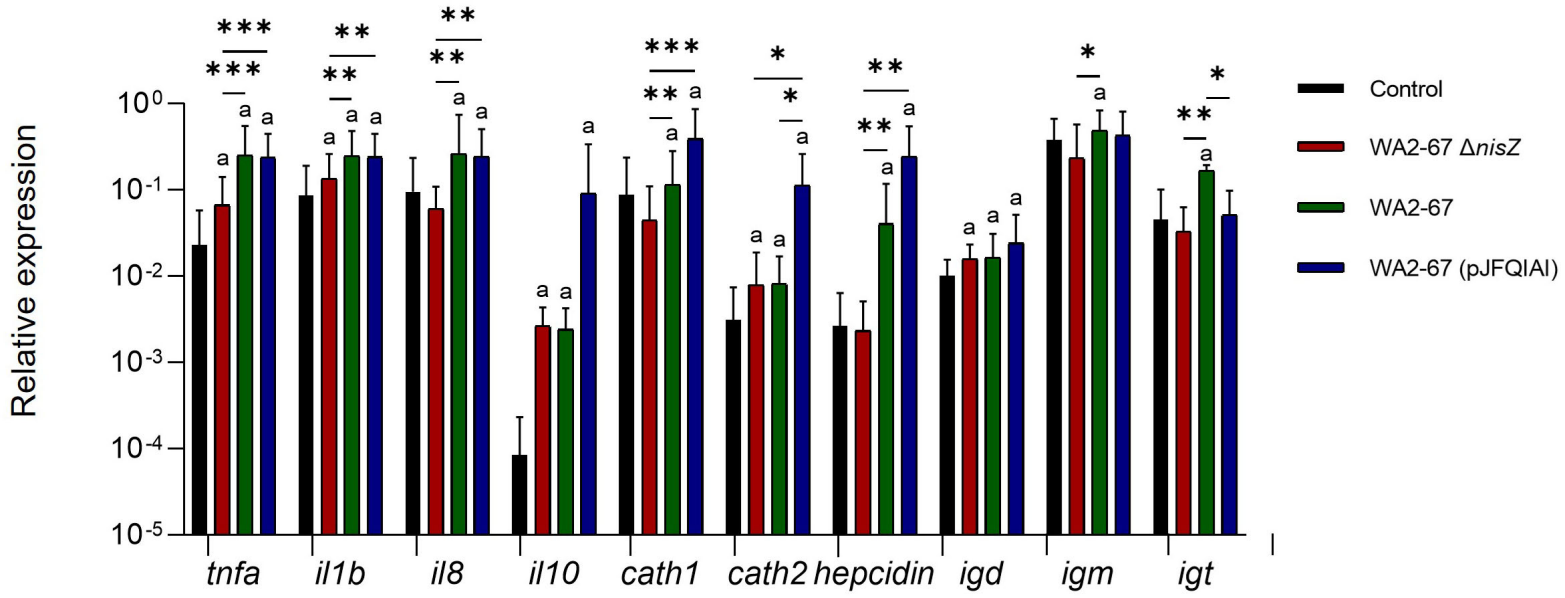
Transcriptional response of splenic leukocytes to different LAB strains: *L. cremoris* WA2-67 Δ*nisZ*, *L. cremoris* WA2-67 and *L. cremoris* WA2-67 (pJFQIAI). Splenic leukocytes were incubated with 1 x 10^6^ cfu/ml of each bacterial strain at 20°C for 24 h. Subsequently, RNA was extracted and the levels of transcription of different genes were analyzed by real-time PCR. Relative gene expression levels were normalized to the transcription of the housekeeping gene *bactin*. Data are shown as mean fold change + SD (n = 8). The letter (a) represents transcription levels significantly different than those observed in cells not exposed to bacterial strains (control), while the asterisks indicate levels significantly different between bacterial treatments as indicated (*p ≤ 0.05, **p ≤ 0.01 and ***p ≤ 0.001).

We also analyzed the transcriptional effects of the different LAB strains on splenic leukocytes. In this regard, we determined the transcriptional levels of pro-inflammatory cytokines (*tnfa*, *il1b*, and *il8*), an anti-inflammatory cytokine (*il10*), three AMPs (*cathelicidins 1* and *2*, and *hepcidin*), and of three immunoglobulins (*igd, igm*, and *igt*). Although all LAB provoked an increased transcription of both *il1b* and *tnfa*, the transcriptional levels reached in response to the two bacteriocinogenic strains were significantly higher than those provoked by the non-bacteriogenic one (**Figure 3**). The two bacteriocinogenic strains significantly upregulated the transcription of another pro-inflammatory cytokine (*il8*), at levels significantly higher than those found in either untreated cells or in cells exposed to the non-bacteriocinogenic *L. cremoris* strain (**Figure 3**). All three LAB significantly induced the transcription of the anti-inflammatory cytokine *il10* and *igd* in comparison to control untreated cells with no significant differences between them (**Figure 3**). In contrast, only the wild type nisin Z-producing *L. cremoris* WA2-67 significantly upregulated the transcription of *igm* or *igt* (**Figure 3**). Regarding the effects the LAB had on AMP transcription, only the bacteriocinogenic *L. cremoris* strains were capable of significantly upregulating *hepcidin* and *cathelicidin 1* at levels significantly higher than those reached by untreated cells or cells exposed to the non-bacteriocinogenic strain (**Figure 3**). In the case of *cathelicidin 2*, although all LAB significantly increased its mRNA levels when compared to untreated cells, the levels reached in response to the multi-bacteriocinogenic strain were significantly higher than those of the cells exposed to other LAB strains (**Figure 3**).

### 3.4 Effect of the bacterial strains on splenic IgM^+^ B cell survival

**Figure 4:**
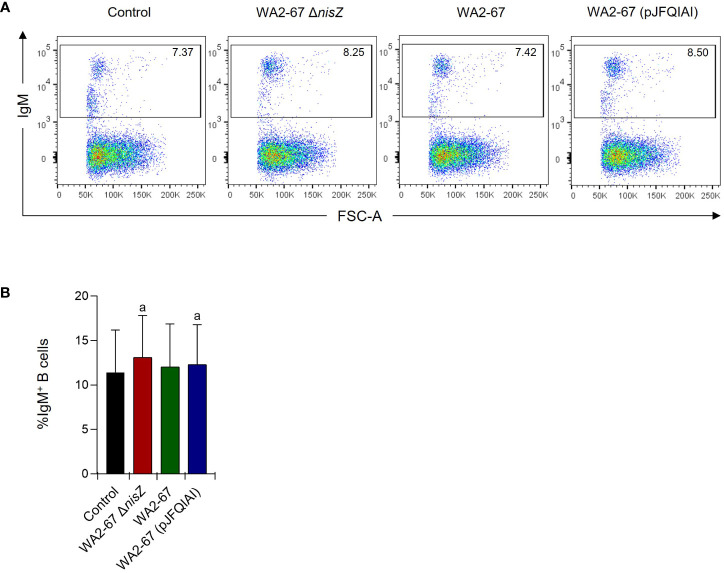
Effects of *L. cremoris* WA2-67 Δ*nisZ*, *L. cremoris* WA2-67, and *L. cremoris* WA2-67 (pJFQIAI) strains on the percentage of splenic IgM^+^ B cells. The percentage of IgM^+^ B cells was measured via flow cytometry using a specific anti-IgM in spleen leukocyte cultures treated with 1 x 10^6^ cfu/ml of each bacterial strain for 48 h. Controls not treated with bacteria were also included. Representative dot plots **(A)** are included along with a graph **(B)** showing the percentage of live IgM^+^ B cells in the lymphocyte gate (mean + SD; n=5). The letter (a) represents transcription levels significantly different than those observed in cells not exposed to bacterial strains (control).

We next determined how the different LAB strains affected the percentage of IgM^+^ B cells in splenocyte cultures by flow cytometry. After 48 h of incubation, although the percentage of IgM^+^ B cells was higher in all cultures exposed to bacteria, when compared to those found in control cultures, the differences were only significant in response to the multi-bacteriocinogenic and the non-bacteriocinogenic strains (**Figure 4**). Nevertheless, no statistically significant differences were found between the different bacterial treatments (**Figure 4**).

### 3.5 Proliferative effects of the bacterial strains on splenic IgM^+^ B cells

**Figure 5:**
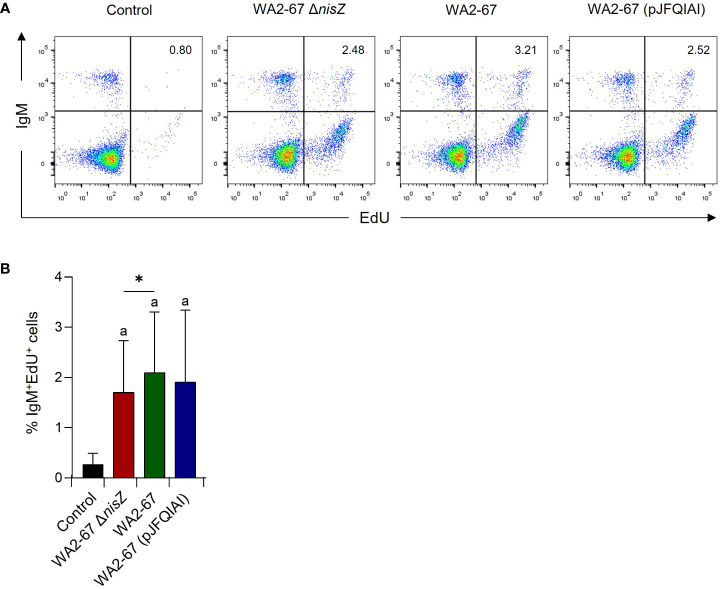
The lymphoproliferative effects of *L. cremoris* WA2-67 Δ*nisZ*, *L. cremoris* WA2-67 and *L. cremoris* WA2-67 (pJFQIAI) on B cells were determined by flow cytometry. For this, cells were stimulated with the bacteria at 20°C for 72 h, and subsequently splenic leukocytes were incubated with EdU for an additional 24 h. At that point, cells were labelled with anti-trout IgM-PE and the percentage of proliferating cells determined. Controls not treated with bacteria were also included. Representative dot plots **(A)** are included along with a graph **(B)** showing the percentage of live IgM^+^ B cells in the lymphocyte gate (mean + SD; n=9). The letter (a) represents transcription levels significantly different than those observed in cells not exposed to the bacterial strains (control), while the asterisks indicate levels significantly different between bacterial treatments as indicated (*p ≤ 0.05).

After having determined that some LAB strains promoted the survival of IgM^+^ B cells in splenocyte cultures, we studied whether they exerted proliferative effects on these cells. Our results showed that all strains could induce a significant proliferation of IgM^+^ B cells in splenocyte cultures, at levels significantly higher than those observed in cultures not exposed to bacteria (**Figure 5**). Interestingly, the lymphoproliferative effect of the wild type nisin Z-producing strain was significantly higher than that produced by its non-bacteriocinogenic isogenic mutant (**Figure 5**).

### 3.6 Effect of the bacterial strains on the respiratory burst activity of splenocytes

**Figure 6:**
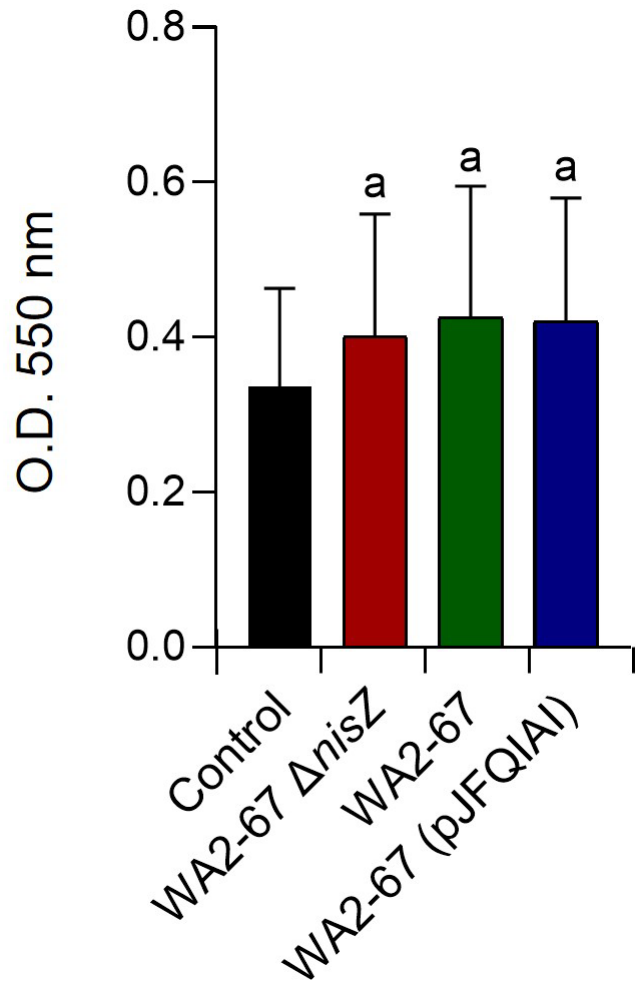
Effects of *L. cremoris* WA2-67 Δ*nisZ*, *L. cremoris* WA2-67 and *L. cremoris* WA2-67 (pJFQIAI) on the respiratory burst activity of splenocytes, via the reduction of the ferricytochrome c by released superoxide anion $\left(O_{2}^{-}\right)$ . Splenocytes were incubated with the different bacterial strains for 30 min at 20°C. Optical density (OD) was then measured at 550 nm. Data are shown as mean OD values + SD (n = 9). The letter (a) represents transcription levels significantly different than those observed in cells not exposed to bacterial strains (control).

We also studied whether the three bacterial strains could trigger the respiratory burst activity of splenocytes. For this, cells were incubated with the different bacteria for 30 min. Our results show that the cells incubated with bacteria released oxygen radicals at levels significantly higher than those observed in cells not exposed to the bacteria (**Figure 6**). Yet, no significant differences were observed between the different bacterial treatments (**Figure 6**).

### 3.7 Effect of the bacterial strains on the NO production of splenocytes

**Figure 7:**
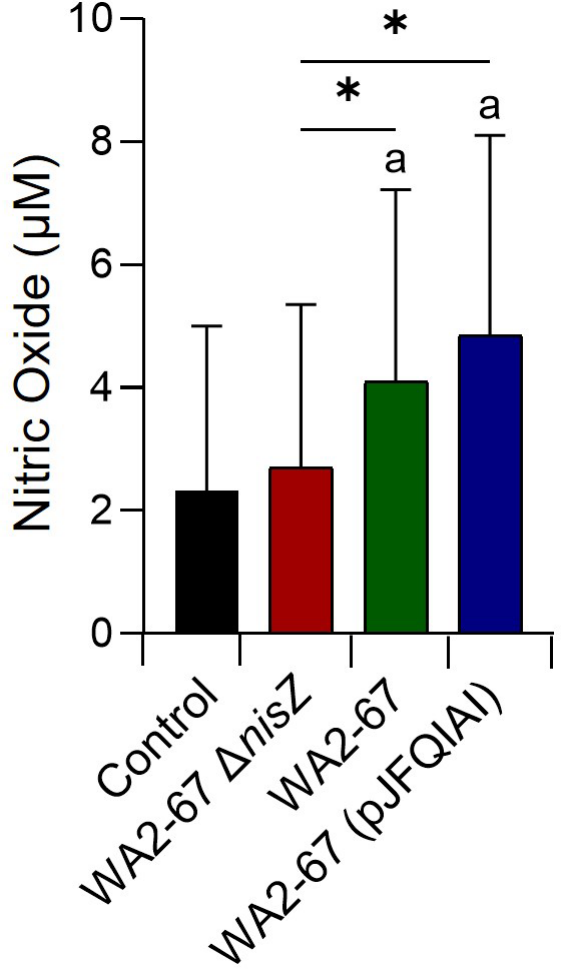
Capacity of *L. cremoris* WA2-67 Δ*nisZ*, *L. cremoris* WA2-67 and *L. cremoris* WA2-67 (pJFQIAI) to induce nitric oxide (NO) production in splenocytes. Cells were incubated at 20°C for 48 h with the different bacterial strains at a concentration of 1 x 10^4^ cfu/ml or with media alone. Subsequently, the optical density (OD) was measured at 540 nm and data readjusted to represent the production of NO in μM. Data are shown as mean values + SD (n = 9). The letter (a) represents NO levels significantly different than those observed in cells not exposed to bacterial strains (control), while asterisks indicate levels significantly different between bacterial treatments as indicated (*p ≤ 0.05).

Finally, we also studied how the different bacterial strains affected the capacity of splenocytes to produce NO. In this case, the cells were incubated for 24 h with the different LAB strains, to then test the accumulation of NO in the supernatants. Interestingly, only the bacteriocinogenic LAB induced the release of NO in splenocytes at levels significantly higher than those reached by untreated cells (**Figure 7**). The NO levels produced by cells in response to the bacteriocinogenic LAB were also significantly higher than the levels produced in response to the non-bacteriocinogenic strain (**Figure 7**).

## 4 Discussion

Over the past years, a growing interest in the use of probiotics as prophylactic agents has emerged in aquaculture as in other animal models. Several of these studies have demonstrated the advantageous immunomodulatory and protective effects of different *Bacillus* spp. and LAB strains on different farmed fish (8–16, 21). Yet, the precise contribution of bacteriocins to the immunomodulatory effects of bacteriocinogenic LAB is still largely unknown in veterinary medicine, as bacteriocins have been traditionally considered immunologically inert peptides. Nonetheless, a few studies have revealed the immunomodulatory capacity of some of these bacteriocins, namely nisin (22–24). Thus, the effect of nisin on cytokine production has been, to some extent, demonstrated in both *in vivo* and *in vitro* experiments, using different mammalian models [reviewed in (25)]. The mechanisms through which bacteriocins exert their immunomodulatory effects are still not clear, yet some authors have proposed that their molecular amphipathic properties, similar to those of AMPs, might explain, to some extent, their common innate immunomodulatory mode of action (24, 25).

Although some contradictory results have been reported, possibly due to the great diversity of the experimental models, a high number of studies have revealed a positive effect of nisin on the production of pro-inflammatory cytokines in mammals (22–25). Nevertheless, to the extent of our knowledge, these effects had never been evaluated in fish before. Therefore, in this work, we investigated the induction of pro-inflammatory cytokines by bacteriocinogenic LAB, using both an intestinal epithelial cell line and splenic leukocytes. Furthermore, rather than stimulating the cells directly with nisin we chose an approach in which the effects of the aquatic wild type nisin Z-producer *L. cremoris* strain were compared to a non-bacteriocinogenic isogenic mutant. Additionally, the effect of a recombinant nisin Z, garvicin A and Q-producer multi-bacteriocinogenic strain was also tested in parallel. The results obtained were more robust in splenic leukocyte cultures, where it was clearly demonstrated that the capacity of the bacteriocinogenic strains to induce *il1b*, *tnfa* and *il8* transcription were significantly higher than those provoked by the non-bacteriocinogenic mutant. Curiously, this difference was only clear for *il8* in the case of RTgutGC cells. Nevertheless, the results are in concordance with the upregulation of pro-inflammatory cytokines such as *tnfa*, *il1b* and *il8*, previously reported by other authors in fish stimulated with probiotic lactococci (26, 27). When regarding the anti-inflammatory cytokine *il10*, it was only in RTgutGC epithelial cells that the bacteriocinogenic strains provoked a significantly higher response than the non- bacteriocinogenic mutant. Interestingly, the regulation of *il10* in the gut in response to different LAB stimuli has already been reported, suggesting that this cytokine is preferentially regulated by probiotic LAB in this tissue. For instance, *il10* transcription was upregulated by *L. lactis* BFE920 in the gut of olive flounder (*Paralichthys olivaceus*) (14). Additionally, Moroni et al. also reported a high upregulation of *il10* transcription in the intestine of gilthead sea bream (*Sparus aurata*) fed with a diet including a high dose of a bacteriocinogenic nisin-producer *L. lactis* (28).

In the current work, we also analyzed the impact of the three LAB strains on the transcriptional levels of different AMPs, namely hepcidin and two cathelicidins. These ancient antimicrobial molecules can act as a host defense mechanism against several pathogens, as well as modulator agents of the host innate immunity. These AMPs can be produced either constitutively or be induced in response to different stimuli, which include inflammatory cytokines produced by other cell types or even microbial products (29–32). Our results revealed that both *cathelicidin 2* and *hepcidin* transcription was preferentially induced by the bacteriocinogenic strains in RTgutGC cells. A similar response was observed for *cathelicidin 1* and *hepcidin* in splenic leukocytes. Curiously, when regarding *cathelicidin 2*, the multi-bacteriocinogenic strain showed the highest stimulatory capacity. Although Lüders et al. already suggested a synergistic effect between LAB bacteriocins and eukaryotic AMPs (33), to the extent of our knowledge, this is the first report on the effects of bacteriocins on the expression of teleostean AMPs. As previously suggested by Lüders et al., this synergy could be a relevant strategy to increase the specific activity and broaden the target-cell range of both bacteriocins and eukaryotic AMPs (33).

In the case of RTgutGC cells, we also evaluated the effects of the different LAB on the transcription of a range of genes related to intestinal barrier function, integrity, and homeostasis. In this case, there was not a clear preferential effect of the bacteriocinogenic strains over the non-bacteriocinogenic mutant. These results suggest that the observed effects are due to intrinsic characteristics of the *L. cremoris* strain, most likely not associated with bacteriocinogenic properties. However, there was an observable difference in the case of the *imuc* gene, involved with the synthesis of intestinal mucus. This observation could suggest that nisin positively influences the production of intestinal mucus, which can be an additional probiotic trait, which has been often associated with probiotic *Lactococcus* spp. in fish (34). Finally, there were no significant differences between the adherence capacity of the three LAB strains, pointing towards a non-bacteriocinogenic intrinsic capacity of the bacteria.

In the past, increased serum levels of IgM have been reported in fish in response to LAB and other probiotic stimuli (35–38). Thus, to establish the potential contribution of the different bacteriocins to this effect, we studied the effect of the different bacterial strains on the percentage of IgM^+^ B cells and on their proliferation in splenocyte cultures. Surprisingly, although all probiotic LAB strains increased the percentage of splenic IgM^+^ B cells without significant differences between them, the differences when compared to untreated cultures were only significant in the case of the cells treated with the multi-bacteriocinogenic bacteria or with the non-bacteriocinogenic strain. However, in the case of IgM^+^ B cell proliferation, there seemed to be a clear effect of nisin. Thus, although all LAB strains significantly induced the proliferation of splenic IgM^+^ B cells, the levels reached by the wild type strain were significantly higher than those provoked by the non-bacteriocinogenic mutant, supporting a specific effect of nisin on IgM^+^ B cell proliferation.

One of the few reports that tested the direct effects of nisin on fish leukocytes was that conducted by Villamil et al. in turbot (*Scophthalmus maximus*, L.) (39). This work revealed a positive effect of nisin on the chemiluminescent response of turbot head-kidney macrophages, which is associated with the respiratory burst activity. Hence, we decided to investigate whether these effects were also visible in rainbow trout. However, in this work, a direct effect of nisin on the respiratory burst did not seem evident, as all three LAB strains, regardless of their bacteriocinogenic properties, had a similar capacity to trigger the respiratory burst of splenic leukocytes. However, this effect has not been yet assessed in rainbow trout head-kidney leukocytes, which represents an interesting line for further investigation for our group. Furthermore, our observations on the NO production by leukocytes upon bacteriocinogenic stimulation also diverge from those of Villamil et al. (39). Although Villamil et al. did not observe an induction of NO production by turbot head-kidney macrophages after nisin stimulation (39), we observed that the capacity of the nisin-producing *L. cremoris* strains to induce NO production was significantly higher than that of the non-bacteriocinogenic mutant strain. Interestingly, when the levels of transcription of the inducible NO synthase (iNOS) were evaluated in splenocyte cultures exposed to the different bacterial strains, we found that iNOS transcription levels were always very low in splenocytes, and consequently no significant differences were found among groups (data not shown). This seems to point to a very specific leukocyte subset being responsible for NO production in response the bacteriocinogenic bacterial strains. Nonetheless, these results constitute the first description of NO production in fish in response to bacteriocinogenic probiotics. Of course, it might be possible that this is not a direct effect but rather induced by the production and expression of the TNF-α, which is a well-known stimulator of NO production, both in fish and mammals (40–43).

In conclusion, to establish the immunomodulatory effects of LAB bacteriocins, we have compared the effects of a wild type nisin-expressing *L. cremoris* strain to those elicited by a non-bacteriocinogenic isogenic mutant and a recombinant nisin Z, garvicin A and Q-producer multi-bacteriocinogenic strain. Our results demonstrated that the bacteriocinogenic strains had a superior capacity to induce the transcription of *il8*, *il10*, *hepcidin* and *imuc* in RTgutGC cells. Nevertheless, the binding capacity to this cell line was similar for the three strains. On the other hand, regarding splenic leukocytes, the bacteriocinogenic strains induced *tnfa*, *il1b, il8*, *cathelicidin 1* and *hepcidin* transcription at levels significantly higher than those provoked by the non-bacteriocinogenic strain. In these cases, as no differences were found between the effects of the two bacteriocinogenic LAB, the production of additional garvicins do not seem to have a differentiated immunomodulatory effect. However, in the case of *cathelicidin 2*, the multi-bacteriocinogenic strain provoked significantly higher effects, possibly pointing to some garvicin-related effects in the regulation of this gene. Additional experiments performed to establish the effects of the different strains on splenic IgM^+^ B cells point towards a capacity of nisin to induce the proliferation of these cells. Finally, although no differences were found among the capacity of the different strains to induce a respiratory burst activity, the bacteriocinogenic strains showed a superior capacity to trigger NO production in splenocyte cultures. All these data further contribute to establish the immunomodulatory potential of bacteriocins, namely nisin, in teleost fish.

## Data availability statement

The original contributions presented in the study are included in the article/**Supplementary Materials**, further inquiries can be directed to the corresponding author/s.

## Ethics statement

The animal study was reviewed and approved by INIA Ethics Committee.

## Author contributions

DC performed all the experiments with the help of FD and PD-R. PD-R, RS, JF, EM-A, JB, and LD-F helped DC to analyze the data and to obtain the figures. LC, CT, PH, and PP conceived the work and designed the experiments. DC wrote the main body of the paper with the help of CT, LC, PH, and PP. All authors contributed to the article and approved the submitted version.
