# Supplementary material for: Immunomodulatory effects of bacteriocinogenic and non-bacteriocinogenic Lactococcus cremoris of aquatic origin on rainbow trout (Oncorhynchus mykiss, Walbaum)
Source: Front Immunol. 2023 Apr 20;14:1178462. doi: 10.3389/fimmu.2023.1178462 (PMC10159052; doi:10.3389/fimmu.2023.1178462)
Supplement: Supplementary file 1 [file Table_1.pdf]

Table S1. List of primers used for real-time PCR in this study.

Gene	Forward primer	Reverse primer
ef1a	GATCCAGAAGGAGGTCACCA	TTACGTTCGACCTTCCATCC
bactin	TCCTTCCTCGGTATGGAGTCT	TTACGGATGTCCACGTCACAC
tnfa	CCACACACTGGGCTCTTCTT	GTCCGAATAGCGCCAAATAA
il1b	GACATGGTGCGTTTCCTTTT	ACCGGTTTGGTGTAGTCCTG
il8	ATTGAGACGGAAAGCAGACG	CTTGCTCAGAGTGGCAATGA
il10	CTGCTGGACGAAGGGATTCTAC	GGCCTTTATCCTGCATCTTCTC
cath1	ACCAGCTCCAAGTCAAGACTTTGAA	TGTCCGAATCTTCTGCTGCAA
cath2	ACATGGAGGCAGAAGTTCAGAAG	GAGCCAAACCCAGGACGAGA
hepcidin	GCTGTTCCTTTCTCCGAGGT	GTGACAGCAGTTGCAGCAC
igd	AGCTACATGGGAGTCAGTCAACT	CTTCGATCCTACCTCCAGTTCCT
igm	TGCGTGTTTGAGAACAAAGC	GACGGCTCGATGATCGTAAT
igt	AACATCACCTGGCACATCAA	TTCAGGTTGCCCTTTGATTC
cdh1	ACTATGACGAGGAGGGAGGT	TGGAGCGATGTCATTACGGA
cldn3	AGGCAACGACGCTACATCAA	GAAACCCAAGCAATGCGTCA
zo1	GCTGTTCCTCCTAGACCTT	TCACCCACATCTGACTCTAC
villin	AGAGCGGTCTGAGTCTTT	GCACCATCATTCACCATCT
imuc	TCAACACATTCTCTGACACC	GGCAGTTACTGTACCAAGTC
